# Superior sensitive graphene fiber sensor enabled by constructing multiple nanoembossments for glucose detection

**DOI:** 10.1038/s41378-025-00903-9

**Published:** 2025-03-17

**Authors:** Feng Han, Yangguang Wu, Yifan Zhao, Weixuan Jing, Kun Zheng, Chenying Wang, Song Wang, Yaxin Zhang, Tao Dong, Zhuangde Jiang

**Affiliations:** State Key Laboratory for Manufacturing Systems Engineering, International Joint Laboratory for Micro/Nano Manufacturing and Measurement Technologies, School of Instrument Science and Technology, Xi’an Jiaotong University, Xi’an, China

**Keywords:** Nanoscience and technology, Nanosensors

## Abstract

Metal oxides have been extensively investigated in non-enzymatic biosensors for detecting diabetes owing to their electrochemical catalytic properties and excellent stability. However, lower conductivity and catalytic activity are major obstacles to the commercialization of metal oxide-based non-enzymatic glucose sensors. Herein, we present a novel flexible nonenzymatic glucose sensor utilizing graphene fiber (GF)/Au/Ni(OH)_2_ composite fiber. The integration of GFs enables a significant uptake of sensing molecules due to its expansive surface area and high electron mobility, ultimately resulting in a decrease in the detection limit. Consequently, the incorporation of Ni(OH)_2_ provides abundant attachment sites by introducing Au atoms, thereby promoting electron migration and enhancing sensitivity and detection limits. An impressive sensitivity (1095.63 µA mM^−1^ cm^−2^) within the detection range (5 µM–2.2 mM) of the integrated GF/Au/Ni(OH)_2_ fiber is achieved, leading to an incredibly low detection limit (0.294 µM). Additionally, the outstanding repeatability, anti-interference properties, and flexibility of the GF/Au/Ni(OH)_2_ sensors are obtained as well. Our findings offer a novel method for constructing nano embossments on GFs to achieve superior glucose detection capabilities in the field of wearable electronics in the future.

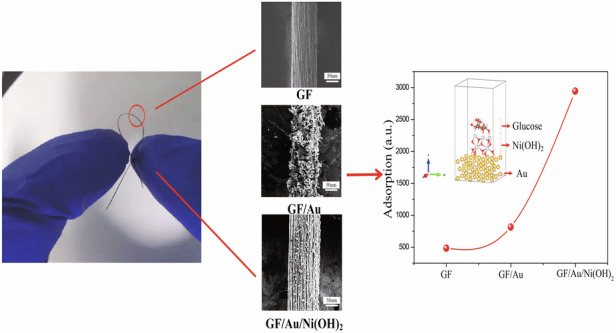

## Introduction

Diabetes constitutes a prominent chronic noncommunicable disease that poses a threat to global human health. The accurate monitoring of blood glucose levels is indispensable for the prevention and treatment of diabetes^1,2^. Over recent decades, diverse strategies for glucose detection have been proposed, including fluorescence, electrochemistry, spectrometry, and chemiluminescence^3,4^. Among them, electrochemical glucose sensors have attracted widespread attention due to their exceptional sensitivity, low detection limit, low cost, and fast response^5^. Moreover, electrochemical sensors can be categorized as either enzyme-based or non-enzyme-based^6–9^. In contrast to enzyme electrochemical biosensors, non-enzyme-based biosensors offer advantages, such as higher electrical conductivity and environmental stability at a lower cost^10–12^. Therefore, non-enzymatic electrochemical biosensors hold great potential as a candidate for glucose detection^13–16^.

The core component of glucose sensors of this type typically consists of a nonenzymatic electrode, comprising a substrate electrode and catalyst materials^17,18^. The substrate electrodes of nonenzymatic glucose sensors have been gradually miniaturized, resulting in lightweight and portability^19,20^. Among them, fiber-shaped nonenzymatic glucose sensors (FNGSs) have gained significant attention owing to their outstanding flexibility, lightweight, high signal-to-noise ratio, and increased current density^21,22^. Fiber glucose sensors play a critical role in wearable technology and other fields because of their small size and convenient wearing^23,24^. However, the primary challenge in fabricating FNGSs lies in developing flexible fiber electrodes with superior conductivity. Traditional conducting fibers such as metal fibers^25^ and carbon fibers^26^ are limited by the low specific surface area^27^. Conversely, metal oxides can serve as catalyst materials (Co_3_O_4_, CuO, CuO_2_, ZnO, Ni (OH)_2_, Co(OH)_2_, etc.), which are extensively studied for nonenzymatic glucose sensing in alkaline environments^28^.

However, the commercialization of metal oxide-based nonenzymatic glucose sensors is hindered by low conductivity and catalytic activity^29^. Recently, there has been witnessed a remarkable upsurge in the eagerness for exploring the synergistic effect of metal–metal oxide (MMO) heterostructures to enhance electrocatalytic performance^30,31^. MMOs form metal-supported interfaces and strong coupling interactions, promoting catalyst stability and electron migration^32–36^. Therefore, it is of paramount importance to design a sensor with a large specific surface area and the synergistic effect of MMO heterostructures for detecting glucose in accordance with the requirements of low detection limits and high sensitivity.

In our work, a novel flexible nonenzymatic glucose sensor is shown as a GF/Au/Ni(OH)_2_ composite fiber. The graphene fibers (GFs) are utilized to support a high uptake of sensing molecules due to their expansive surface area and outstanding electron mobility, thereby contributing to a reduction in the detection limit^37–41^. Additionally, the implementation of Au atoms into Ni(OH)_2_ provides abundant attachment sites for enhanced electron migration between the working electrode and Ni(OH)_2_, ultimately leading to improved sensitivity and lower detection limits^42^. The integrated GF/Au/Ni(OH)_2_ electrode manifests an impressive sensitivity (1095.63 µA mM^−1^ cm^−2^) within the detection range (5 µM–2.2 mM), resulting in an ultra-low detection limit (0.294 µM). The extraordinary morphologies of nano embossments are constructed through the electrodeposition of Au and Ni(OH)_2_ to form MMO heterojunctions on the surface of GFs, providing multiple active sites at various angles for efficient electron migration channels. Furthermore, the GF/Au/Ni(OH)_2_ sensors also demonstrate excellent repeatability, anti-interference properties, and flexibility. As the flexible nonenzymatic glucose sensor, our findings offer a novel approach for constructing nano embossments on GFs to achieve superior glucose detection capabilities in the future.

## Results and discussion

**Fig. 1:**
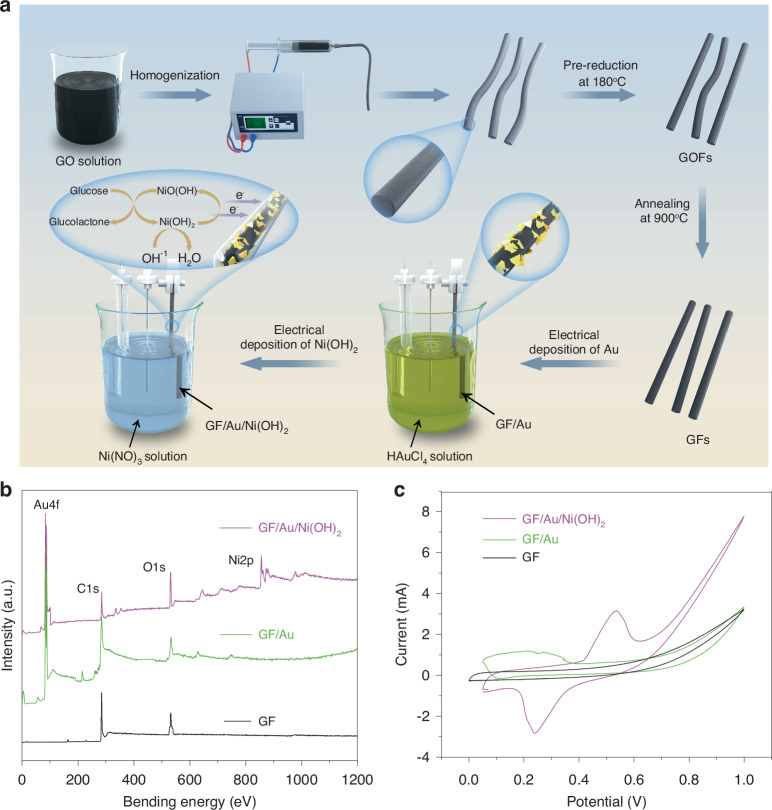
**a** Manufacturing process of GF/Au/Ni(OH)_2_ electrode. **b** XPS spectra of GF, GF/Au, and GF/Au/Ni(OH)_2_. **c** CV curves of GF, GF/Au, GF/Au/Ni(OH)_2_ composite electrode in 0.1 M NaOH solution with glucose

Figure 1a illustrates the fabrication process of GF/Au/Ni(OH)_2_ fiber, which involves a three-step method including microfluidic spinning, thermal annealing, and electrochemical deposition, respectively. Initially, the homogeneous GO solution is spun using microfluidic spinning and pre-reduced at 180 °C to form GOFs. Subsequently, the resultant GOFs are further reduced at a temperature of 900 °C under inert gas protection to yield GFs. Finally, GFs are converted into GF/Au/Ni(OH)_2_ through electrochemical deposition. X-ray photoelectron spectroscopy (XPS) analysis is carried out to identify the chemical and electronic configurations of multiple elements in the GF, GF/Au, and GF/Au/Ni(OH)_2_ composite fiber electrodes, as respectively, illustrated in Fig. 1b. The survey spectra unveil the presence of C1*s* (284 eV) and O1*s* (532 eV) in all three film samples, while characteristic peaks of Au4*f* and Ni2*p* are observed in GF/Au and GF/Au/Ni(OH)_2_, respectively (Fig. S1a, b), indicating the deposition of Au and Ni(OH)_2_ onto the surface of GF is successfully achieved. The electrochemical properties of the GF, GF/Au, and GF/Au/Ni(OH)_2_ fibers are probed through cyclic voltammetry (CV) tests in a conventional three-electrodes system. The CV curves of GF, GF/Au, and GF/Au/Ni(OH)_2_ under identical conditions are depicted in Fig. 1c. Upon investigation, it is ascertained that the graphene shows no redox peak within the working potential range, indicating its intrinsically electrochemically inert. Notably, an oxidation peak at 0.2 V is evident in the CV curves recorded for the GF/Au composite electrode. This observation can be attributed to the increase in the number of AuOH_ads_ sites on the GF/Au electrode when reaching 0.2 V, subsequently leading to catalytic glucose oxidation and a consequent increase in current. In the further, the GF/Au/Ni(OH)_2_ electrode displays a reduction peak at 0.24 V and an oxidation peak at 0.58 V, which can be ascribed to the presence of Ni (II)/Ni(III) redox couple. It is worth noting that the nanoscale Ni(OH)_2_ undergoes oxidation to NiOOH and reverts back to Ni(OH)_2_ through potential cycling in alkaline solutions. Figure S2a–c reveals the CV curves of the GF, GF/Au, and GF/Au/Ni(OH)_2_ fiber electrodes in 0.1 M NaOH solution with/without glucose, respectively. It is found that the GF/Au and GF/Au/Ni(OH)_2_ fiber electrodes have a favorable catalytic effect on glucose.

**Fig. 2:**
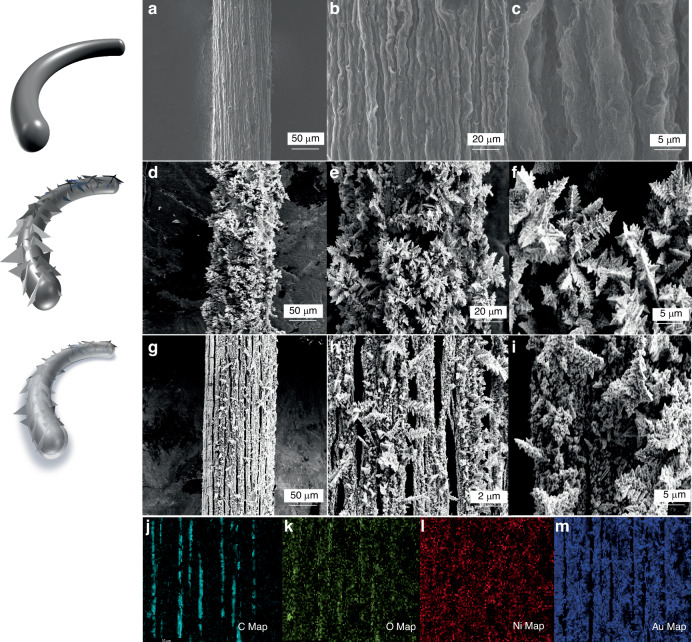
The structural morphology of the electrode surface. **a**–**c** The GF surface at different magnifications; **d**–**f** The GF/Au surface at different magnifications; **g**–**i** GF/Au/Ni(OH)_2_ surface at different magnification; **j**–**m** shows the EDS element mapping of GF/Au/Ni(OH)_2_

The surface morphology of the resultant GF, GF/Au, and GF/Au/Ni(OH)_2_ composite fibers are examined through scanning electron microscopy (SEM) method. Figure 2a–c depicts rough and wrinkled surface of GF with a diameter of ~130 μm, which offers a larger specific surface area for subsequent Au deposition. After the deposition of Au on the surface of GF (Fig. 2d–f), nano embossments are formed on the surface, increasing the fiber diameter to nearly 150 μm and further enhancing its specific surface area as an electrode material. Additionally, the typical images of GF/Au/Ni(OH)_2_ are shown in Fig. 2g–i, respectively. The Ni(OH)_2_ with a thickness of 2 µm is uniformly distributed on the surface of GF/Au composite fiber electrodes. The deposition of Ni(OH)_2_ leads to the flattening of nano embossments generated by Au, supporting multiple active sites for electron migration channels at various angles. The structural evolution diagram of the fiber electrode is clearly illustrated on the left of Fig. 2. As shown in Fig. 2j–m, the mapping conducted by energy dispersive spectrometer mapping (EDS) demonstrates the existence of C, Au, Ni, and O. The distribution of element C is confined to local gaps while Au, Ni, and O are dispersed throughout all regions, indicating successful deposition of Au and Ni (OH)_2_ onto the GF surface.

**Fig. 3:**
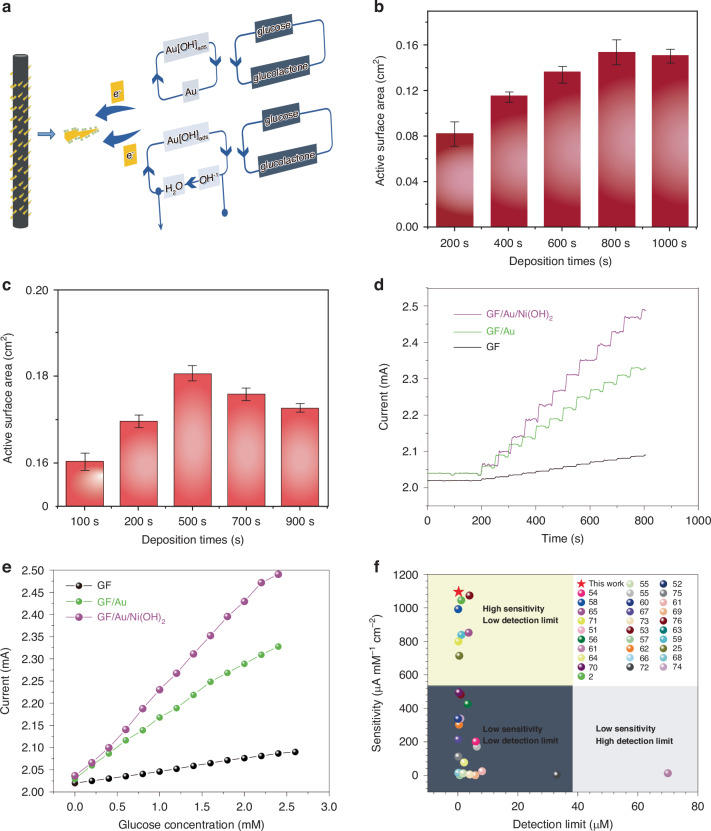
**a** Catalytic mechanism of GF/Au/Ni(OH)_2_ composite fiber electrode. **b** The electrochemically active surface areas of GF/Au composite fiber with different deposition times. **c** The electrochemical active surface areas of GF/Au/ Ni(OH)_2_ composite fiber with different deposition. **d** The *I–t* curves of the GF, GF/Au, GF/Au/Ni(OH)_2_ composite electrode with successive addition of glucose in 0.1 M NaOH solution. **e** The corresponding calibration curve of the GF, GF/Au, GF/Au/Ni(OH)_2_ composite fiber electrode. **f** Comparison of the analytical performance of our proposed GF/Au/Ni(OH)_2_ sensor with others

The mechanism of electrocatalytic oxidation of glucose on GF/Au/Ni(OH)_2_ composite fiber is illustrated in Fig. 3a. Hydroxide anions are chemisorbed on the Au surface, giving rise to the formation of hydrous gold oxide (AuOH_ads_)^43^, which then interacts with glucose molecules to promote their oxidation into glucolactone^44,45^. Simultaneously, Ni(OH)_2_ undergoes electron transfer through Au/GF, transforming into NiO(OH) and catalyzing the diffusion of glucose onto the electrode surface for rapid oxidation into glucolactone. The specific equation for the catalytic oxidation of glucose by Au and nickel hydroxide can be expressed as follows^46^:

1 $${\rm{Au}}+{\rm{O}}{{\rm{H}}}^{-}\to {\rm{Au}}[{\rm{OH}}]_{{\rm{ads}}}^{{(1-{\lambda })}^{-}}+{\lambda }{{\rm{e}}}^{-}$$

2 $${\rm{Au}}[{\rm{OH}}]_{{\rm{ads}}}^{{(1-{\lambda })}^{-}}+{\rm{glucose}}\to {\rm{Au}}+{\rm{glucolactone}}$$

3 $${\rm{Ni}}({\rm{OH}}{)}_{2}+{\rm{O}}{{\rm{H}}}^{-}\to {\rm{NiO}}\left({\rm{OH}}\right)+{{\rm{e}}}^{-}+{{\rm{H}}}_{2}{\rm{O}}$$

4 $${\rm{NiO}}\left({\rm{OH}}\right)+{\rm{glucose}}\to {\rm{Ni}}({\rm{OH}}{)}_{2}+{\rm{glucolactone}}$$

The ads and *λ* represent the chemically adsorbed species on GF/Au and the partial charge-transfer coefficient, which ranges from 0 to 1^47^. Additionally, the MMO composed of Au and Ni(OH)_2_ demonstrates a synergistic effect among its individual components: Ni(OH)_2_ can inhibit the absorption of poisoning species on the surfaces of GF/Au, while the GF/Au can facilitate enhanced electron migration.

To compare the electrochemical active surface area of GF/Au and GF/Au/Ni(OH)_2_ electrodes deposited at different durations, CV curves are achieved in a solution containing Fe(CN)_6_^4−/3−^ (5 mM) and KCl (0.1 M) at the specific rate of (25 mV s^−1^) in Fig. S3a, b. The electrochemical surface area (ECSA) of GF/Au and GF/Au/Ni(OH)_2_ composite fibers exhibit a gradual increase with the extension of deposition time as well. It suggests that the ECSA of GF/Au and GF/Au/Ni(OH)_2_ electrodes can reach values of 0.164 and 0.186 cm^2^ (Fig. 3b, c), respectively, as determined by using the following Randles Sevcik equation^48,49^:

$${{I}}_{{\rm{p}}}=2.69\times {10}^{5}{A}{{D}}^{1/2}{{n}}^{3/2}{{v}}^{1/2}{C}$$

where *I*_p_ means the peak current intensity, *n* denotes the amounts of electrons involved in the redox process, *A* stands for ECSA (cm^2^), *D* means the diffusion coefficient (7.6 × 10^−6^ cm^2^ s^−1^), *C* represents the concentration of electroactive species (5 × 10^−6^ mol cm^3^), and *v*^1/2^ (V s^−1^) signifies the square root of scan rate.

Figure 3d displays the amperometric curves of current-time in GF, GF/Au, and GF/Au/Ni(OH)_2_ fibers with sequential additions of glucose. The modified fiber demonstrates a rapid amperometric response, achieving a steady-state current within <5 s upon the addition of glucose to the stirred support electrolyte (Fig. S4). The calibration curve depicted in Fig. 3e showcases the remarkable linear dependence of the GF/Au/Ni(OH)_2_ fiber within a glucose concentration range from 5 µM to 2.2 mM, bringing about an impressive correlation coefficient (0.9979), a sensitivity (1095.63 µA mM^−1^ cm^−2^), and a detection limit (0.294 µM), respectively.

Figure 3f illustrates the superior performance of our non-enzymatic glucose fiber sensor in contrast to similar sensors^2,25,50–76^, showcasing a detection limit ((0.294 µM) and sensitivity (1095.63 µA mM^−1^ cm^−2^). These results highlight the outstanding overall performance and accuracy of our glucose sensor in detecting glucose. Further comparison with other non-enzyme glucose sensors listed in Table S1, the core–shell structure of carbon nanotubes is utilized to form the glucose sensors. It increases the special surface area and metal-organic frameworks for enhancing the electrocatalytic performance and sensitivity. On the other hand, the sensitivity of our fabricated glucose sensor exceeds that of Ni and Ni(OH)_2_-based sensors by a simpler preparation process. The remarkable electrochemical properties are ascribed to the unique composition of Ni(OH)_2_ and Au in MMO, providing an abundance of catalysis sites for glucose oxidization, thereby optimizing detection limit, sensitivity, and linear range. Additionally, direct deposition of Ni(OH)_2_ and Au on GF accelerates electron migration between oxidation sites and the fiber surface, while the extraordinary conductivity and minuscule size of a single GF contribute to rapid response times

**Fig. 4:**
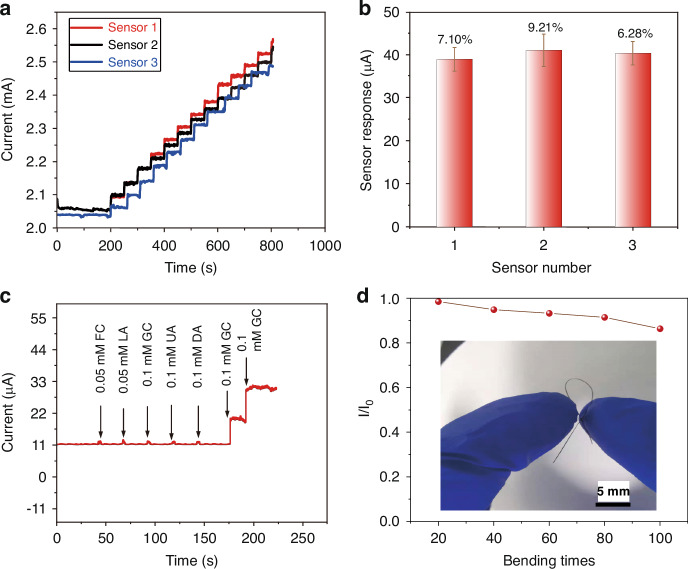
**a** Amperometric response of three different GF/Au/Ni(OH)_2_ composite fiber electrodes with successive addition of 0.2 mM glucose. **b** The mean and standard deviation of the responses of the three electrodes towards 0.2 mM glucose in 0.1 M NaOH. **c** GF/Au/Ni(OH)_2_ composite fiber electrode response to glucose and some common interferences, FC, LA, GC, UA DA, and GC. **d** Sensitivity of GF/Au/Ni (OH)_2_ composite fiber electrode after bending, inset shows the shape after bending

Reproducibility is a crucial characteristic of sensors for practical application. Therefore, the three fibers were meticulously fabricated and assessed through amperometric measurements in NaOH solution (0.1 M) to investigate the reproducibility of GF/Au/Ni(OH)_2_ fiber. The signal response from three distinct GF/Au/Ni(OH)_2_ fibers in Fig. 4a is illustrated as glucose (0.2 mM) is successively added, ranging from 0 µM to 2.4 mM. Figure 4b presents the trend of the mean and standard deviation (RSD) of electrodes’ responses, revealing a maximum RSD of 9.21% and thus demonstrating excellent reproducibility of GF/Au/Ni(OH)_2_.

In complex physiological environments, the presence of oxidative interfering substances can influence the accuracy of glucose detection. Hence, selectivity is another key property for nonenzymatic glucose sensors in clinical applications. The amperometric response was recorded in the presence of common interfering substances in the blood to explore the selectivity of the GF/Au/Ni(OH)_2_ fiber. The anti-interference test involves sequentially adding 0.1 mM (glucose (GC), Galactose (GC), Uric acid (UA), and Dopamine (DA)) and 0.05 mM (d-Fructose (FC)), Lactate Acid (LA) into NaOH (0.1 M) solution, as shown in Fig. 4c, respectively. It deduces that all the tested foreign species have negligible interference to the glucose signal, indicating the outstanding selectivity of GF/Au/Ni(OH)_2_ electrode towards common coexisting interfering substances in the blood. The GF/Au/Ni(OH)_2_ fiber in our work reveals excellent electrochemical performance and flexibility under diverse deformations. As depicted in Fig. 4(d), the GF/Au/Ni(OH)_2_ also maintains a sensitivity of 91.4% after 80 deformation cycles, emphasizing its superior flexibility. Additionally, we conducted fiber performance following 100 bending cycles at angles ranging from 30° to 150° (30°, 60°, 90°, 120°, and 150°) in Fig. S5. When the fiber is bent at an angle below 90°, its performance remains essentially unchanged, while it can still retain 91.5% of its performance when bent within the range of 120–150°. Our findings reveal that the fibers exhibit excellent performance even under low-angle bending, with significant degradation only observed beyond 120° (Table S2). Consequently, the GF/Au/Ni(OH)_2_ electrode holds great potential for various applications.

**Fig. 5:**
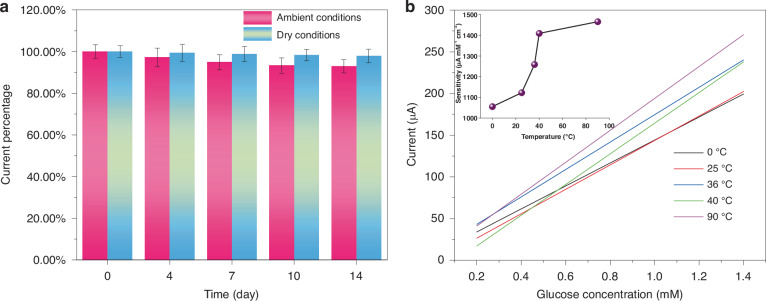
**a** Current response variation of 1 mM glucose on GF/Au/Ni(OH)_2_ electrode versus storage time. **b** Corresponding calibration curves of the GF/Au/Ni(OH)_2_ composite fiber electrode At 0, 25, 36, 40, and 90 °C, respectively

The enduring stability of GF/Au/Ni(OH)_2_ fiber is assessed by monitoring the current response of glucose (1 mM) over a period of 14 days at room temperature and in a dry environment (Fig. S6). As depicted in Fig. 5a, although there is a slight decrease in the response current, it still maintains a robust performance (92.9%) under continuous testing for 14 days. The impact of humidity storage conditions on fiber sensor properties has been determined to be significant (Fig. S7). Stability tests carried out under dry storage conditions indicate that the fiber sensor can retain 97.9% of its performance after 14 days of storage. Consequently, the GF/Au/Ni(OH)_2_ electrode exhibits excellent stability during prolonged storage. On the other hand, glucose detection is not limited to a single temperature. Therefore, it is essential to test glucose sensors at various temperatures. As illustrated in Fig. 5b, the GF/Au/Ni(OH)_2_ fiber is subjected to glucose testing at 0, 25, 36, 40, and 90 °C, respectively. It can be deduced that the GF/Au/Ni(OH)_2_ fiber sensor maintains exceptional sensitivity in detecting glucose across a broad spectrum of temperatures, including both low and high extremes.

**Fig. 6:**
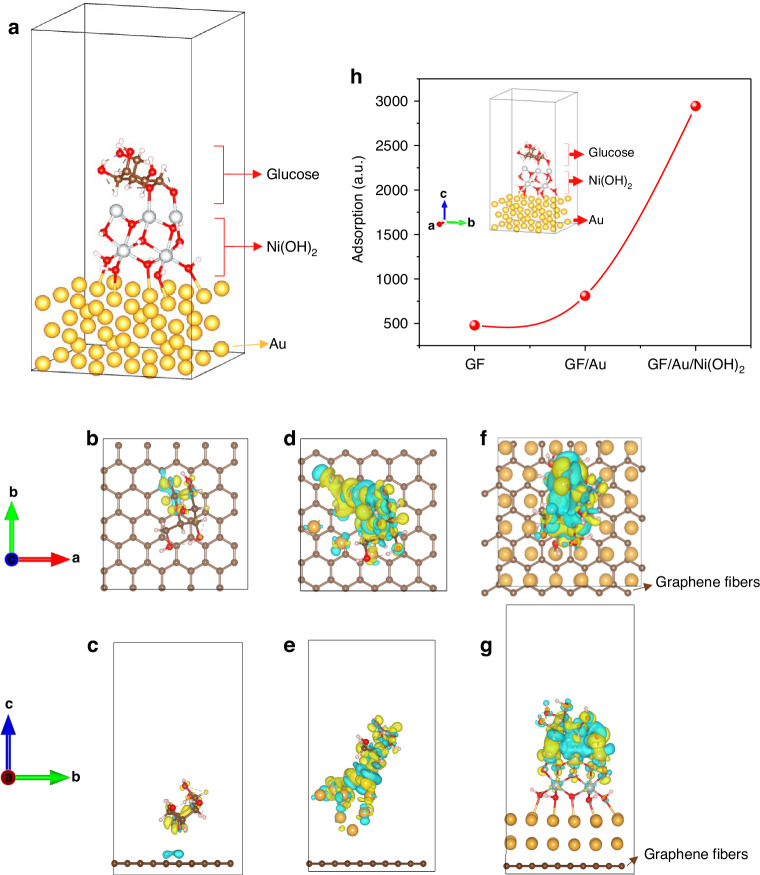
The calculated model of **a** GF/Au/Ni(OH)_2_ with glucose. **b** and **c** GF sensor with glucose, **d** and **e** GF/Au sensor with glucose, and **f** and **g** GF/Au/Ni(OH)_2_ with glucose, respectively. **b**, **d**, and **f** sensor models are in the vertical angle of view and **c**, **e**, **g** sensor models are in the horizontal angle of view. **h** The calculated adsorptions of glucose using the fiber sensors with a different structure

The model of the GF/Au/Ni(OH)_2_ sensor has been established, as depicted in Fig. 6a. The molecules of Glucose, Ni(OH)_2_, and Au atoms are illustrated as well, respectively. Through the utilization of modeling and mechanism analysis, we have unequivocally validated the reliability of our experimental findings regarding the properties of fiber sensors. The establishment of calculation methods utilized in developing the theoretical model can be outlined meticulously in Supporting Information. In Fig. 6, it is visually presented that the yellow cloud-like portion within the model signifies electron aggregation, while blue represents electron depletion. This serves to more intuitively illustrate the differential charge transfer process. Two-dimensional section combined with atomic coordinates is used to present the data form. Specifically, through several stages including GF adsorption, GF/Au adsorption, and GF/Au/Ni(OH)_2_ adsorption, a significant charge transfer phenomenon between glucose and Au-loaded Ni(OH)_2_ substrate has been observed in Fig. 6f and g. It expounds that GF/Au/Ni(OH)_2_ actively participates in glucose adsorption and enhances sensor sensitivity, leading to the consequences shown in Fig. 6h. Our combination of modeling and experimental validation has effectively unveiled the crucial role of Au-loaded Ni(OH)_2_ in glucose adsorption for sensor applications, offering valuable insights for optimizing sensor performance.

We employ a glucose molecule as the adsorption model. Given that calculating adsorption capacity in DFT is challenging, most studies on adsorption typically focus on the size of adsorption using a single molecule. Based on the EDD outcomes and adsorption energy, it appears that there’s a greater electron transfer between the Ni(OH)_2_-loaded model and glucose. Specifically, Ni in Ni(OH)_2_ seems to be capable of donating more electrons to the oxygen atoms in glucose, leading to higher adsorption energy. In contrast, Au and graphene surface carbon atoms exhibit less electron transfer to the oxygen in glucose. To further validate this observation, the Muliken charges^77,78^ were computed, which were obtained through wave function analysis with the assistance of Multiwfn software for glucose adsorption on three distinct substrates as presented in Table S4. The wave function modulus files before and after adsorption were calculated, and the Muliken charge was determined using Multiwfn to obtain the amount of electron transfer amount by subtraction. The findings reveal that the electron transfers between GF, GF/Au, GF/Au/Ni(OH)_2_, and glucose are 0.17e, 0.34e, and 0.42e, respectively. Consequently, we propose that the Ni(OH)_2_-loaded model favors glucose adsorption.

## Conclusion

In summary, a novel flexible GF/Au/Ni(OH)_2_ composite fiber has been developed by electrochemical depositing MMO onto a graphene fiber microelectrode. This composite material combines the remarkable electrocatalytic performance of Ni(OH)_2_ with the prominent conductivity of Au. The resultant fiber-shaped GF/Au/Ni(OH)_2_ fiber electrode exhibits an impressive sensitivity of 1095.63 µA mM^−1^ cm^−2^, along with a remarkably low detection limit of 0.169 µM and a linear range spanning from 5 µM to 2.2 mM. Due to its good reproducibility and flexibility, GF/Au/Ni(OH)_2_ composite fiber has great promising for applications in wearable and implantable sensing.

### Experimental sections

#### Materials and reagents

Graphene oxide (GO) was synthesized using a modified version of Hummer’s method. Chlorauric acid (AuCl_4_), nickel nitrate (Ni(NO_3_)_2_), and sodium hydroxide (NaOH) were obtained from Sinopharm Chemical Reagent Co., Ltd. Glucose (GC), Uric acid (UA), Dopamine (DA), D-Fructose (FC), Galactose (GC) and Lactate Acid (LA) were purchased from Sigma-Aldrich Co., LLC. All aqueous solutions were prepared using deionized (DI) water. The counter electrode (Pt electrode) and reference electrode (KCl solution saturated Ag/AgCl electrode) were purchased from Shanghai Chenhua Instrument Company.

#### Preparation of graphene fibers (GFs)

A simple two-step method was used to produce graphene fibers (GFs), involving microfluidic spinning and thermal annealing. Initially, a well-dispersed solution of graphene oxide (GO) at 12 mg/mL was prepared through ultrasonic treatment for 30 min. The GO solution was then injected into a polytetrafluoroethylene (PTFE) microreactor tube with an internal diameter of 2 mm using a syringe pump at a flow rate of 10 mL/h. After sealing the tube ends, the GO fibers (GOFs) were formed in the microfluidic channel under uniform vapor pressure and heated at 180 °C for 2 h in an oven. Subsequently, the preformed GOFs were pushed out from the tube with N_2_ flow and dried in atmosphere conditions. Then, the GOFs were subjected to high-temperature annealing in a furnace under controlled vacuum and gas flows. Prior to heating, a vacuum atmosphere of 0.1 Pa and ultrapure Argon protective flow of 200 sccm were maintained for 2 h to remove O_2_ and water vapor. The fibers were then annealed under argon protection at a heating rate of 2.5 °C/min up to 900 °C, followed by annealing for another 2 h before cooling to room temperature. Following thermal annealing, the GO fibers were reduced to GFs.

#### Preparation of the GF/Au/Ni(OH)_2_ fiber electrodes

The deposition of Au and Ni(OH)_2_ on GFs electrode was carried out by an electrochemical method. A traditional three-electrodes system was used with a Pt electrode as the counter electrode, an Ag/AgCl electrode as the reference electrode and a single GF electrode as the working electrode, as shown in Fig. 1a. The Au species were electrodeposited from electrolytes of 0.5 M AuCl_4_ by using the amperometric *i*–*t* curve method with the electrodeposition parameters including voltage (−0.2 V) and a series of deposition time (200, 400, 600, 800, 1000). Subsequently, the Au/GF electrode was immersed in another electrolyte solution of Ni(NO_3_)_2_ with the electrodeposition parameters including voltage (−0.4 V) and a series of deposition times (100, 300, 500, 700, 900). After deposition, the GF/Au/Ni(OH)_2_ electrode was carefully washed with DI water and dried at room temperature. Therefore, the GF/Au/Ni(OH)_2_ electrode was prepared.

#### Characterization

Morphology images of fibers were observed using scanning electron microscopy (SEM, Hitachi, SU-8010). Chemical identification was conducted using X-ray photoelectron spectroscopy (XPS, Thermo Fisher Escalab xi+). Raman spectrum analysis was performed with a laser Raman spectrometer (Horiba Jobin Yvon, HR800)

#### Electrochemical tests

The CHI 760D electrochemical workstation was used to conduct electrochemical measurements on fiber microelectrodes, utilizing a three-electrode electrochemical cell. Working electrodes included graphene fiber (GF), GF/Au, or GF/Au/Ni(OH)_2_ microelectrodes, while a platinum wire (0.5 mm diameter) and an Ag/AgCl (KCl, 0.1 M) electrode served as the counter and reference electrodes, respectively. Cyclic voltammetry (CV) and Amperometric *i*–*t* curve (IT) were employed to measure the microelectrodes in a 0.1 M NaOH electrolyte solution. A cyclic voltammogram was performed in a 1 mM glucose solution with a scan rate of 50 mV s^−1^ and a potential range of 0−+1 V. When the background current decayed to a steady state, amperometric response was performed with a 100 μl glucose solution successively added into 50 ml 0.1 M NaOH with bias voltage of +0.8 V. The applied voltage during the test was relative to the Ag/AgCl electrode. Scatter plot was drawn according to the data measured by the current–time method, and linear fitting was carried out to obtain the linear fitting curve. The sensitivity was obtained by dividing the slope of the linear fitting curve by the fiber surface area, and the detection line was obtained by dividing the slope of the linear fitting curve by three times the standard deviation. The pH experiment of the sensor was carried out in 0.1 mM (pH 13), 0.01 mM (pH 12), and 0.001 mM (pH 11) NaOH solutions for IT testing. The temperature of the storage test for the sensor are 0, 25, and 40 °C for one day, respectively. Subsequently, the amperometric response was carried out in a 1 mM glucose solution. The response current is used to determine the impact of storage temperature on the sensor. Similarly, the humidity of storage test for the sensor was conducted at 50%, 60%, 70%, and 80% levels for one day, followed by the amperometric response in a 1 mM glucose solution. The response current is used to determine the impact of storage humidity on the sensor

## Supplementary information

Supplemental Material

## References

[1] Wei, C. et al. Metal organic framework-derived anthill-like Cu@carbon nanocomposites for nonenzymatic glucose sensor [J]. *Anal. Methods* **6**, 1550–1557 (2014).

[2] Balakrishnan, S. R. et al. Development of highly sensitive polysilicon nanogap with APTES/GOx based lab-on-chip biosensor to determine low levels of salivary glucose [J]. *Sens. Actuators A-Phys.* **220**, 101–111 (2014).

[3] Yang, J. X. et al. Highly efficient microreactors with simultaneous separation of catalysts and products in deep desulfurization [J]. *Chem. Eng. J.* **267**, 93–101 (2015).

[4] Lu, P. et al. Synthesis and characterization of nickel oxide hollow spheres-reduced graphene oxide-nafion composite and its biosensing for glucose [J]. *Sens. Actuators B-Chem.* **208**, 90–98 (2015).

[5] Zhang, D. J. et al. 3D porous metal-organic framework as an efficient electrocatalyst for nonenzymatic sensing application [J]. *Talanta* **144**, 1176–1181 (2015). 26452944 10.1016/j.talanta.2015.07.091

[6] Huang, J. W. et al. A novel glucose sensor based on MoS_2_ nanosheet functionalized with Ni nanoparticles [J]. *Electrochim. Acta* **136**, 41–46 (2014).

[7] Wang, L. et al. One-step synthesis of Pt–NiO nanoplate array/reduced graphene oxide nanocomposites for nonenzymatic glucose sensing [J]. *J. Mater. Chem. A* **3**, 608–616 (2015).

[8] Wang, L. et al. Electrochemical sensing and biosensing platform based on biomass-derived macroporous carbon materials [J]. *Anal. Chem.* **86**, 1414–1421 (2014). 24422469 10.1021/ac401563m

[9] Wang, L. et al. Simple and large-scale strategy to prepare flexible graphene tape electrode [J]. *ACS Appl. Mater. Interfaces* **9**, 9089–9095 (2017). 28222258 10.1021/acsami.6b14624

[10] Wang, L. et al. Nickel–cobalt nanostructures coated reduced graphene oxide nanocomposite electrode for nonenzymatic glucose biosensing [J]. *Electrochim. Acta* **114**, 484–493 (2013).

[11] Xu, W. N. et al. Nanorod-aggregated flower-like CuO grown on a carbon fiber fabric for a super high sensitive non-enzymatic glucose sensor [J]. *J. Mater. Chem. B* **3**, 5777–5785 (2015). 32262574 10.1039/c5tb00592b

[12] Chang, G. et al. Synthesis of highly dispersed Pt nanoclusters anchored graphene composites and their application for non-enzymatic glucose sensing [J]. *Electrochim. Acta* **157**, 149–157 (2015).

[13] Li, G. L. et al. Dual structural design of platinum-nickel hydrogels for wearable glucose biosensing with ultrahigh stability [J]. *Small* **19**, 2206868 (2023). 10.1002/smll.202206868 36710247

[14] Zhu, J. et al. Laser-induced graphene non-enzymatic glucose sensors for on-body measurements [J]. *Biosens. Bioelectron.* **193**, 113606 (2021). 34507206 10.1016/j.bios.2021.113606 PMC8556579

[15] Luo, Y. et al. Integration of triphenylene-based conductive metal-organic frameworks into carbon nanotube electrodes for boosting nonenzymatic glucose sensing [J]. *ACS Appl. Mater. Interfaces* **15**, 51435–51443 (2023). 10.1021/acsami.3c11810 37903405

[16] Zhao, Y. M. et al. Boosting electrochemical catalysis and nonenzymatic sensing toward glucose by single-atom Pt supported on Cu@CuO core–shell nanowires [J]. *Small* **19**, 2207240 (2023). 10.1002/smll.202207240 36703531

[17] Burke, L. D. Pemonolayer oxidation and its role in electrocatalysis [J]. *Electrochim. Acta* **39**, 1841–1848 (1994).

[18] Hsiao, M. W., Adzic, R. R. & Yeager, E. B. Electrochemical oxidation of glucose on single crystal and polycrystalline gold surfaces in phosphate buffer [J]. *J. Electrochem. Soc.* **143**, 759–767 (1996).

[19] Yang, Y. R. & Gao, W. Wearable and flexible electronics for continuous molecular monitoring [J]. *Chem. Soc. Rev.* **48**, 1465–1491 (2019). 29611861 10.1039/c7cs00730b

[20] Lu, Y. et al. Wearable sweat monitoring system with integrated micro-supercapacitors [J]. *Nano Energy* **58**, 624–632 (2019).

[21] Liu, Y. et al. Highly sensitive detection of hydrogen peroxide at a carbon nanotube fiber microelectrode coated with palladium nanoparticles [J]. *Microchim. Acta* **181**, 63–70 (2014).

[22] Wang, L. et al. PtAu alloy nanoflowers on 3D porous ionic liquid functionalized graphene-wrapped activated carbon fiber as a flexible microelectrode for near-cell detection of cancer [J]. *NPG Asia Mater.* **8**, e337 (2016).

[23] Li, Q. F. et al. Pt/MXene-based flexible wearable non-enzymatic electrochemical sensor for continuous glucose detection in sweat. *ACS Appl. Mater. Interfaces* **15**, 13290–13298 (2023). 36862063 10.1021/acsami.2c20543

[24] Lorestani, F. et al. A highly sensitive and long-term stable wearable patch for continuous analysis of biomarkers in sweat [J]. *Adv. Funct. Mater.* **33**, 2306117 (2023). 10.1002/adfm.202306117 PMC10959519 38525448

[25] Sanford, A. L. et al. Voltammetric detection of hydrogen peroxide at carbon fiber microelectrodes. *Anal. Chem.* **82**, 5205–5210 (2010). 10.1021/ac100536s PMC2902973 20503997

[26] Billon, G. & van den Berg, C. M. G. Gold and silver micro-wire electrodes for trace analysis of metals [J]. *Electroanalysis* **16**, 1583–1591 (2004).

[27] Chen, Y. H. et al. A wearable non-enzymatic sensor for continuous monitoring of glucose in human sweat [J]. *Talanta* **278**, 126499 (2024). 10.1016/j.talanta.2024.126499 38968652

[28] Dong, Q. C., Ryu, H. & Lei, Y. Metal oxide based non-enzymatic electrochemical sensors for glucose detection [J]. *Electrochim. Acta* **370**, 137744 (2021).

[29] Zhu, H. et al. Advances in non-enzymatic glucose sensors based on metal oxides [J]. *J. Mater. Chem. B* **4**, 7333–7349 (2016). 32263734 10.1039/c6tb02037b

[30] Li, L. et al. Synergetic photocatalytic and thermocatalytic reforming of methanol for hydrogen production based on Pt@TiO_2_ catalyst [J]. *Chin. J. Catal.* **43**, 1258–1266 (2022).

[31] Lu, H. J. et al. Photoelectrocatalytic hydrogen peroxide production based on transition-metal-oxide semiconductors [J]. *Chin. J. Catal.* **43**, 1204–1215 (2022).

[32] Meng, C. et al. Atomically and electronically coupled Pt and CoO hybrid nanocatalysts for enhanced electrocatalytic performance [J]. *Adv. Mater.* **29**, 1604607 (2017). 10.1002/adma.201604607 28026056

[33] Li, R. C. et al. New TiO_2_-based oxide for catalyzing alkaline hydrogen evolution reaction with noble metal-like performance [J]. *Small Methods* **5**, 2100246 (2021). 10.1002/smtd.202100246 34927904

[34] Wei, J. X. et al. In situ confining Pt clusters in ultrathin MnO_2_ nanosheets for highly efficient hydrogen evolution reaction [J]. *Small Struct.* **2**, 2100047 (2021).

[35] Zaman, S. et al. Oxygen reduction electrocatalysts toward practical fuel cells: progress and perspectives [J]. *Angew. Chem.-Int. Ed.* **60**, 17832–17852 (2021). 10.1002/anie.202016977 33533165

[36] Niu, H. T. et al. Rational design and synthesis of one-dimensional platinum-based nanostructures for oxygen-reduction electrocatalysis [J]. *Chin. J. Catal.* **43**, 1459–1472 (2022).

[37] El-Kady, M. F. et al. Laser scribing of high-performance and flexible graphene-based electrochemical capacitors [J]. *Science* **335**, 1326–1330 (2012). 22422977 10.1126/science.1216744

[38] Liu, C. G. et al. Graphene-based supercapacitor with an ultrahigh energy density [J]. *Nano Lett.* **10**, 4863–4868 (2010). 21058713 10.1021/nl102661q

[39] Miller, J. R., Outlaw, R. A. & Holloway, B. C. Graphene double-layer capacitor with ac line-filtering performance [J]. *Science* **329**, 1637–1639 (2010). 20929845 10.1126/science.1194372

[40] Yang, X. et al. A high-performance graphene oxide-doped ion gel as gel polymer electrolyte for all-solid-state supercapacitor applications [J]. *Adv. Funct. Mater.* **23**, 3353–3360 (2013).

[41] Zhang, L. et al. Porous 3D graphene-based bulk materials with exceptional high surface area and excellent conductivity for supercapacitors [J]. *Sci. Rep.* **3**, 1408 (2013). 23474952 10.1038/srep01408 PMC3593215

[42] Chen, J. & Zheng, J. B. A highly sensitive non-enzymatic glucose sensor based on tremella-like Ni(OH)_2_ and Au nanohybrid films [J]. *J. Electroanal. Chem.* **749**, 83–88 (2015).

[43] Li, X. Y. & Du, X. Z. Molybdenum disulfide nanosheets supported Au–Pd bimetallic nanoparticles for non-enzymatic electrochemical sensing of hydrogen peroxide and glucose [J]. *Sens. Actuators B-Chem.* **239**, 536–543 (2017).

[44] Huang, Y. et al. A strategy for the formation of gold–palladium supra-nanoparticles from gold nanoparticles of various shapes and their application to high-performance H_2_O_2_ sensing [J]. *J. Phys. Chem. C* **119**, 26164–26170 (2015).

[45] Velasco-Garcia, M. N. & Mottram, T. Biosensor technology addressing agricultural problems [J]. *Biosyst. Eng.* **84**, 1–12 (2003).

[46] Wang, J. G. et al. Electrochemical oxidation and determination of glucose in alkaline media based on au (111)-like nanoparticle array on indium tin oxide electrode [J]. *Electrochim. Acta* **138**, 174–186 (2014).

[47] Kannan, P., Sampath, S. & John, S. A. Direct growth of gold nanorods on gold and indium tin oxide surfaces: spectral, electrochemical, and electrocatalytic studies [J]. *J. Phys. Chem. C* **114**, 21114–21122 (2010).

[48] Luo, Y. et al. One-pot preparation of reduced graphene oxide-carbon nanotube decorated with Au nanoparticles based on protein for non-enzymatic electrochemical sensing of glucose [J]. *Sens. Actuators B-Chem.* **234**, 625–632 (2016).

[49] Liu, Y. et al. Nonenzymatic glucose sensor based on renewable electrospun Ni nanoparticle-loaded carbon nanofiber paste electrode [J]. *Biosens. Bioelectron.* **24**, 3329–3334 (2009). 19450966 10.1016/j.bios.2009.04.032

[50] Hoa, L. T., Sun, K. G. & Hur, S. H. Highly sensitive non-enzymatic glucose sensor based on Pt nanoparticle decorated graphene oxide hydrogel [J]. *Sens. Actuators B-Chem.* **210**, 618–623 (2015).

[51] Ismail, N. S. et al. Development of non-enzymatic electrochemical glucose sensor based on graphene oxide nanoribbon—gold nanoparticle hybrid [J]. *Electrochim. Acta* **146**, 98–105 (2014).

[52] Gao, X. J. et al. Core-shell gold-nickel nanostructures as highly selective and stable nonenzymatic glucose sensor for fermentation process [J]. *Sci. Rep.* **10**, 1365 (2020). 31992829 10.1038/s41598-020-58403-x PMC6987199

[53] Pasta, M., La Mantia, F. & Cui, Y. Mechanism of glucose electrochemical oxidation on gold surface [J]. *Electrochim. Acta* **55**, 5561–5568 (2010).

[54] Guo, M. M. et al. Ultrasensitive nonenzymatic sensing of glucose on Ni(OH)_2_-coated nanoporous gold film with two pairs of electron mediators [J]. *Electrochim. Acta* **142**, 351–358 (2014).

[55] Ayranci, R. et al. Use of the monodisperse Pt/Ni@rGO nanocomposite synthesized by ultrasonic hydroxide assisted reduction method in electrochemical nonenzymatic glucose detection [J]. *Mater. Sci. Eng. C Mater. Biol. Appl* **99**, 951–956 (2019). 30889769 10.1016/j.msec.2019.02.040

[56] Shu, Y. et al. Highly stretchable wearable electrochemical sensor based on Ni–Co MOF nanosheet-decorated Ag/rGO/PU fiber for continuous sweat glucose detection [J]. *Anal. Chem.* **93**, 16222–16230 (2021). 34813294 10.1021/acs.analchem.1c04106

[57] Meng, W. et al. A novel electrochemical sensor for glucose detection based on Ag@ZIF-67 nanocomposite [J]. *Sens. Actuators B: Chem.* **260**, 852–860 (2018).

[58] Adeniyi, O. et al. Nanohybrid electrocatalyst based on cobalt phthalocyanine-carbon nanotube-reduced graphene oxide for ultrasensitive detection of glucose in human saliva [J]. *Sens. Actuators B: Chem.* **348**, 130723 (2021).

[59] Zhou, J. et al. Layered assembly of NiMn-layered double hydroxide on graphene oxide for enhanced non-enzymatic sugars and hydrogen peroxide detection [J]. *Sens. Actuators B: Chem.* **260**, 408–417 (2018).

[60] Jia, H. et al. Facile preparation of Ni nanoparticle embedded on mesoporous carbon nanorods for non-enzymatic glucose detection [J]. *J. Colloid Interface Sci.* **583**, 310–320 (2021). 33007587 10.1016/j.jcis.2020.09.051

[61] Pal, N., Banerjee, S. & Bhaumik, A. A facile route for the syntheses of Ni(OH)_2_ and NiO nanostructures as potential candidates for non-enzymatic glucose sensor [J]. *J. Colloid Interface Sci.* **516**, 121–127 (2018). 29367062 10.1016/j.jcis.2018.01.027

[62] Shu, Y. et al. Facile synthesis of ultrathin nickel–cobalt phosphate 2D nanosheets with enhanced electrocatalytic activity for glucose oxidation [J]. *ACS Appl. Mater. Interfaces* **10**, 2360–2367 (2018). 29293318 10.1021/acsami.7b17005

[63] Fang, Q. et al. Cu aerogels with sustainable Cu(I)/Cu(II) redox cycles for sensitive nonenzymatic glucose sensing [J]. *Adv. Healthc. Mater.* **12**, e2301073 (2023). 37285868 10.1002/adhm.202301073

[64] Dong, L. et al. In-situ synthesis of Pt nanoparticles/reduced graphene oxide/cellulose nanohybrid for nonenzymatic glucose sensing [J]. *Carbohydr. Polym.* **303**, 120463 (2023). 36657845 10.1016/j.carbpol.2022.120463

[65] Zhao, Y. et al. Boosting electrochemical catalysis and nonenzymatic sensing toward glucose by single-atom Pt supported on Cu@CuO core–shell nanowires [J]. *Small* **19**, e2207240 (2023). 36703531 10.1002/smll.202207240

[66] Franceschini, F. et al. MBE grown vanadium oxide thin films for enhanced non‐enzymatic glucose sensing [J]. *Adv. Funct. Mater.* **33**, 2304037 (2023).

[67] Shi, X. et al. Complete glucose electrooxidation enabled by coordinatively unsaturated copper sites in metal-organic frameworks [J]. *Angew. Chem. Int. Ed. Engl.* **62**, e202316257 (2023). 37941302 10.1002/anie.202316257

[68] Gopalan, A. I. et al. A novel multicomponent redox polymer nanobead based high performance non-enzymatic glucose sensor [J]. *Biosens. Bioelectron.* **84**, 53–63 (2016). 26584775 10.1016/j.bios.2015.10.079

[69] Lee, W. C. et al. Comparison of enzymatic and non-enzymatic glucose sensors based on hierarchical Au-Ni alloy with conductive polymer [J]. *Biosens. Bioelectron.* **130**, 48–54 (2019). 30731345 10.1016/j.bios.2019.01.028

[70] Zhang, Y. et al. A flexible non-enzymatic glucose sensor based on copper nanoparticles anchored on laser-induced graphene [J]. *Carbon* **156**, 506–513 (2020).

[71] Yin, H., Bai, X. & Yang, Z. Activating Ni nanoparticles into Ni single atoms by N doping for high-performance electrochemical sensing of glucose [J]. *Chem. Eng. J.* **478**, 147510 (2023).

[72] Ye, J.-S. et al. Diameter effect of electrospun carbon fiber support for the catalysis of Pt nanoparticles in glucose oxidation [J]. *Chem. Eng. J.* **283**, 304–312 (2016).

[73] Shekarchizadeh, H., Kadivar, M. & Ensafi, A. A. Rapid nonenzymatic monitoring of glucose and fructose using a CuO/multiwalled carbon nanotube nanocomposite-modified glassy carbon electrode [J]. *Chin. J. Catal.* **34**, 1208–1215 (2013).

[74] Das, D. et al. Phosphine-free avenue to Co_2_P nanoparticle encapsulated N,P co-doped CNTs: a novel non-enzymatic glucose sensor and an efficient electrocatalyst for oxygen evolution reaction [J]. *Green Chem.* **19**, 1327–1335 (2017).

[75] Bao, L. et al. 3D graphene frameworks/Co_3_O_4_ composites electrode for high-performance supercapacitor and enzymeless glucose detection [J]. *Small* **13**, 1602077 (2017). 10.1002/smll.201602077 27862948

[76] Zhou, H. et al. Amorphous intermediate derivative from ZIF-67 and its outstanding electrocatalytic activity [J]. *Small* **16**, e1904252 (2020). 31821688 10.1002/smll.201904252

[77] Lu, T. & Chen, F. W. Multiwfn: A multifunctional wavefunction analyzer [J]. *J. Comput. Chem.* **33**, 580–592 (2012). 22162017 10.1002/jcc.22885

[78] Lu, T. A comprehensive electron wavefunction analysis toolbox for chemists, Multiwfn [J]. *J. Chem. Phys.* **161**, 082503 (2024). 10.1063/5.0216272 39189657

