# Supplementary material for: Superior sensitive graphene fiber sensor enabled by constructing multiple nanoembossments for glucose detection
Source: Microsyst Nanoeng. 2025 Mar 17;11:48. doi: 10.1038/s41378-025-00903-9 (PMC11914602; doi:10.1038/s41378-025-00903-9)
Supplement: Supplementary file 1 — Supplemental Material [file 41378_2025_903_MOESM1_ESM.docx]

**Supporting information**

**Superior Sensitive Graphene Fiber Sensor Enabled by Constructing Multiple Nanoembossments for** **Glucose Detection**

Feng Han^1,#^, Yangguang Wu^1,#^, Yifan Zhao^1,^*, Weixuan Jing^1,^*, Kun Zheng^1^, Chenying Wang^1^, Song Wang^1^, Yaxin Zhang^1^, Tao Dong^1^, Zhuangde Jiang^1^

1. State Key Laboratory for Manufacturing Systems Engineering, International Joint Laboratory for Micro/Nano Manufacturing and Measurement Technologies, School of Instrument Science and Technology, Xi’an Jiaotong University, Xi’an, China.

E-mails: zhaoyifan100@xjtu.edu.cn; wxjing@mail.xjtu.edu.cn

#These authors contributed equally to this work

**Keywords:** Fiber sensors; Glucose detection; High sensitivity; Flexible nonenzymatic glucose sensor

**1. XPS and Roman spectra of GF, GF/Au and GF/Au/Ni(OH)_2_ fiber sensors**

The high-resolution spectrum of Au4f (FigS1(a)) reveals the binding energies of bimodal Au4f_7/2_ and Au4f_5/2_ are 84.1ev and 87.5 ev respectively, which indicates that the main form of Au was simple substance. As depicted in Fig.S1(b), the spectrum shows the expected signal peaks of Ni2p_3/2_ (856.0 ev) and Ni2p_1/2_(873.6 ev). The binding energy of Ni2p_3/2_ is different from Ni (852.6ev), NiO (853.7 ev), and NiS (853.1 ev), but similar to Ni(OH)_2_ (855.6 ev). It is evident that the nickel in the synthesized composite exists in the form of Ni(OH)_2_. Raman spectroscopy is performed to characterize the carbon materials in the GF, GF/Au, and GF/Au/Ni(OH)_2_ composite fiber electrodes as shown in Fig.S1(c) respectively. All the composite fibers have distinct peaks of D and G bands originated from graphene lattice, which are located at 1360 and 1630 cm^-1^ respectively. The peaks of D band are arised from the structural defects of disordered carbon, while the peaks of G band can be attributed to ordered sp^2^-hybridized carbon, and the structural disorders of graphitic materials can be appraised quantitatively by the peak intensity ratio I_D_/I_G_. It is evident that I_D_/I_G_ decreases from 1.10 for GF to 0.992 for GF/Au, due to the enhancement of G band intensity caused by the surface enhanced Raman scattering (SERS) effect of Au directly adsorbed on GF. Au form a direct physical connection with the surface of GF, which can reduce the impedance between the electrode surface and the catalytic material and increase the electron transfer rate. Morever, I_D_/I_G_ is slightly decreased to 0.975 after depositing Ni(OH)_2_ for GF/Au, indicating that Ni(OH)_2_ tend to be formed on the defect sites of GF/Au, thereby suppressing the vibrations of D band and leads to reduced I_D_/I_G_.


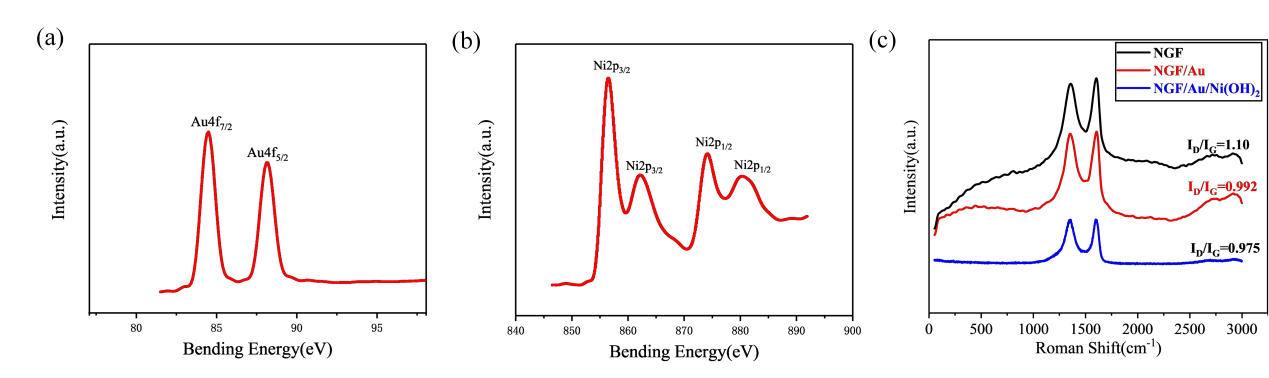


Fig.S1. (a) Au 4f spectra of GF/Au; (b) XPS spectra of Ni2p3 of GF/Au/Ni(OH)_2_; (c) Raman spectra of GF, GF/Au and GF/Au/Ni(OH)_2_.

**2. Cyclic voltammogram curves of GF, GF/Au and GF/Au/Ni(OH)_2_ fiber sensors**

The electrochemical properties of the GF, GF/Au and GF/Au/Ni(OH)_2_ electrodes were studied through CV tests in a traditional three electrodes system. Fig.S2(a-c) reveals CV curves of the GF, GF/Au and GF/Au/Ni(OH)_2_ fiber electrodes in 0.1 M NaOH solution containing glucose and without glucose, respectively. It is found that the GF does not exhibit any redox peak in the working potential range (Fig.S2(a)), indicating that graphene is inherently electrochemically silent. As shown in Fig.S2(b), an oxidation peak is observed at 0.2 V in the CV curves recorded for the GF/Au composite electrode. When the potential reached to 0.2 V, the population of AuOH_ads_ sites on the GF/Au electrode increased and subsequent catalytic oxidation of glucose occurred, which lead to a rise in current. As shown in Fig.S2(c), the GF/Au/Ni(OH)_2_ electrode displays the reduction peak at 0.24 V and oxidation peak at 0.58 V, which is attributed to the presence of Ni (II)/Ni(III) redox couple. Nanoscale Ni(OH)_2_ was oxidized to NiOOH and turned to Ni(OH)_2_ in alkaline solutions by potential cycling.


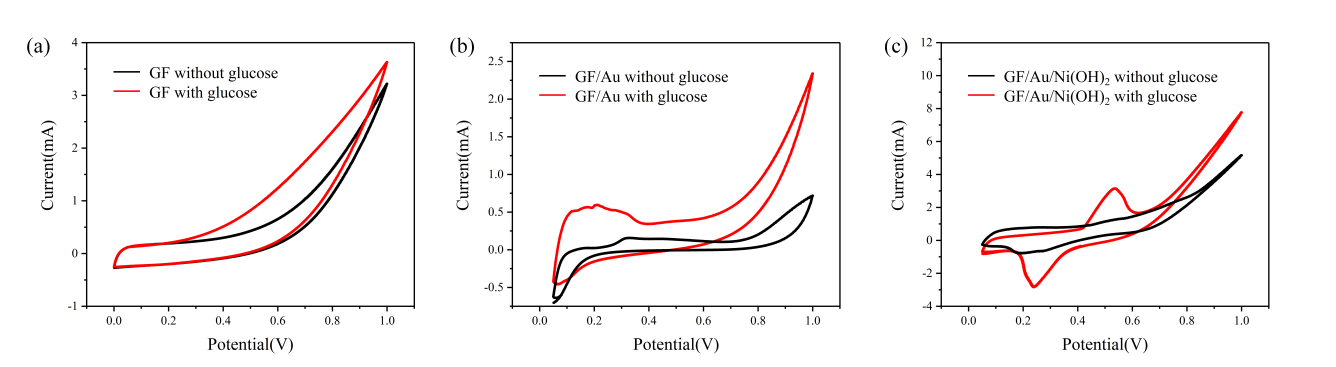


Fig.S2. (a) CV curves of the GF electrode in 0.1 M NaOH solution with and without glucose. (b) CV curves of the GF/Au electrode in 0.1 M NaOH solution with and without glucose. (c) CV curves of the GF/Au/Ni(OH)_2_ electrode in 0.1 M NaOH solution with and without glucose.

In order to compare the electrochemical active surface area of GF/Au and GF/Au/Ni(OH)_2_ electrodes deposited at different times, CV curves are achieved in a solution containing 5 mM Fe(CN)_6_^4-/3-^ with 0.1 M KCl at a scan rate of 25 mV s^-1^. Electrochemical surface area (ECSA) of the GF/Au and GF/Au/Ni(OH)_2_ composite fibers exhibit a gradual increase with prolonged deposition time, as shown in Fig.S3(a,b).


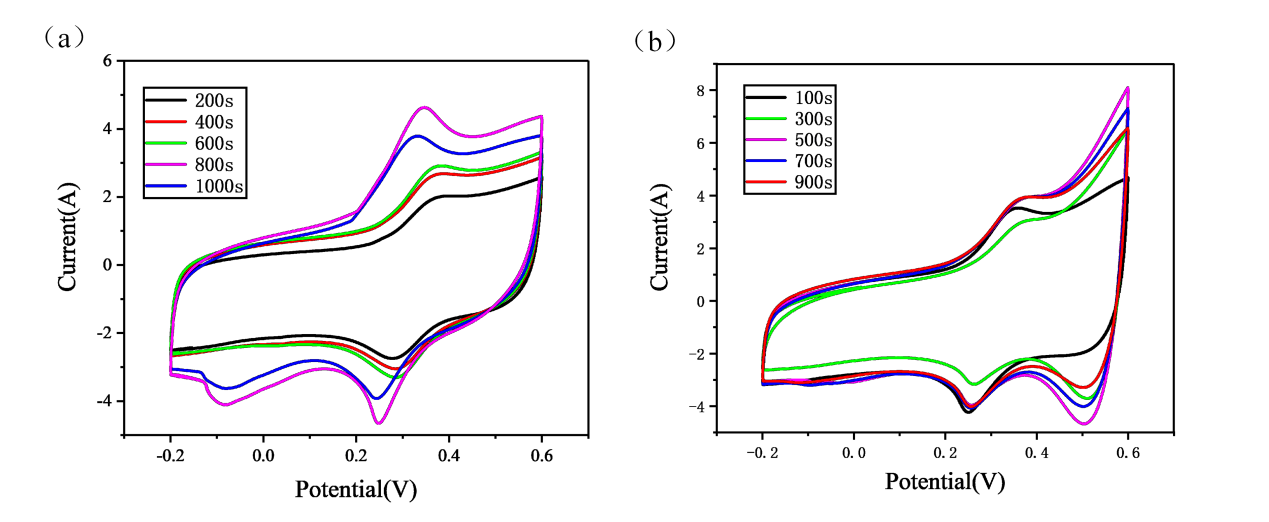


Fig.S3. (a) Cyclic voltammogram curves of the GF/Au composite fiber with different deposition times in 5 mM K_3_Fe(CN)_6_/0.1 M KCl solution at a scan rate of 0.05 V/s; (b) Cyclic voltammogram curves of the GF/Au/Ni(OH)_2_ composite fiber with different deposition times in 5 mM K_3_Fe(CN)_6_/0.1 M KCl solution at a scan rate of 0.05 V/s.

**3. The methods of simulations for GF, GF/Au and GF/Au/Ni(OH)_2_ fiber sensors**

All simulations were carried out using spin-polarized methods as implemented in the QUICKSTEP code of the CP2K 2024 package which based on density functional theory. The general gradient approximation (GGA) parametrized by Perdew, Burke, and Ernzerhof (PBE) was used as the exchange-correlation functional. The Kohn–Sham (KS) equations are solved according to the Gaussian and plane wave (GPW) formalism. Grimme's DFT-D3 correction was adopted to describe the weak van der Waals interaction. The GPW uses Goedecker–Teter–Hutter pseudo potentials to describe the interactions between core and valence electrons, while the valence electron density is represented in terms of Gaussian type orbital (GTO) basis set functions. In particular, we use DZVP-MOLOPT-SR-GTH basis sets for geometry optimization and TZVP-MOLOPT-SR-GTH basis sets for static calculations, and the Brillouin zone integration was sampled using a Monkhorst-Pack special k-point mesh with a resolution of 2π × 0.04 was applied. The convergence criterion for the maximum force was set as 5 × 10^−4^ atomic units. The auxiliary PW basis set, which is needed for the efficient solution of the Poisson's equation in reciprocal space, is truncated at 500 Ry. All Electronic structure and wave function analysis were conducted using Multiwfn software^[1]^.

Table S1 Performance comparison of non-enzymatic glucose sensors.

| Electrode materials | Sensitivity(µA∙mM^−1^∙cm^−2^) | Detection limit (µM) | Linearity range (mM) | Response  Time（s） | selectivity | Measurement environment | Reference |
| --- | --- | --- | --- | --- | --- | --- | --- |
| Pt/Ni@rGO | 171.92 | 6.3 | 0.004–4.5 |  | ascorbic acid (AA),  phenol, ethanol | 0.1M NaOH | (*Materials Science & Engineering C*, 99, 951-956, **(2019)**) |
| Ni−Co MOF/Ag/rGO/PU | 425.9 | 3.28 | 0.01-0.66 |  | DA、LA、NaCl、UA、cysteine(Cys) | 0.1M NaOH | (*Analytical Chemistry*, 93, 16222−16230, **(2021)**) |
| Ni/NCNs-500 | 337.32 | 0.07 | 0.0001–0.5336 | ＜1.6 |  | 0.1M NaOH | (*Journal of Colloid and Interface Science*, 583, 310–320, **(2021)**) |
| Ni(OH)_2_ nano and NiO nanorods | 12.09 & 24.0 | 70 and 8.1 | 0.1–156 and 0.01–83 |  | AA, UA，DA | 0.2 M NaOH | (*Journal of Colloid and Interface Science*, 516, 121–127, **(2018)**) |
| nickel−cobalt phosphate | 302.99 | 0.2 | 2-4470 |  | AA，DA | 0.1M NaOH | (*ACS Applied Materials Interfaces*, 10, 2360-2367, **(2018)**) |
| Ni-MOF | 907.54 |  | 0.0005-2.6655 | ＜3 | DA、AA、UA、NaCl | 0.1M NaOH | (*Advanced Science*, 10, 2304102, **(2023)**) |
| Cu aerogels | 714.3 | 0.48 | 0.001–1 | ＜2 | Cys, AA, UA, DA, KCl, NaCl, and glutathione(GSH) | 0.1M NaOH | (*Advanced Healthcare Materials*, 12, 2301073, **(2023)**) |
| LIO-Ni electrode | 5222 | 3.31 | 0.005−1.1 |  | UA, AA, acetaminophen （AC）, Glutamic acid（GA）, and citric acid（CA） | 0.1M NaOH | (*ACS Applied Nano Materials*, 3, 5260-5270 **(2020)**) |
| Cu@C nanocubes | 2565 | 21.35 | 0.04–40 |  | UA、DA、AA、Nacl 、tryptophan (Try),、glycine (Gly) | 0.4 M NaOH | (*Sensors and Actuators B: Chemical*, 305, 127473 **(2020)**) |
| Ni_3_(HHTP)_2_/CNTs | 4774 | 4.1 | 0.01-3.9 | ＜2 | Urea、DA、NaCl、UA、AA、LA | 0.1M NaOH | (*ACS Applied Materials & Interfaces*, 15(44), 51435-51443, **(2023)**) |
| GF/Au/Ni(OH)_2_ | 1095.63 | 0.169 | 0.005-2.2 | ＜5 | UA、DA、FC、GC、LA | 0.1M NaOH | This work |


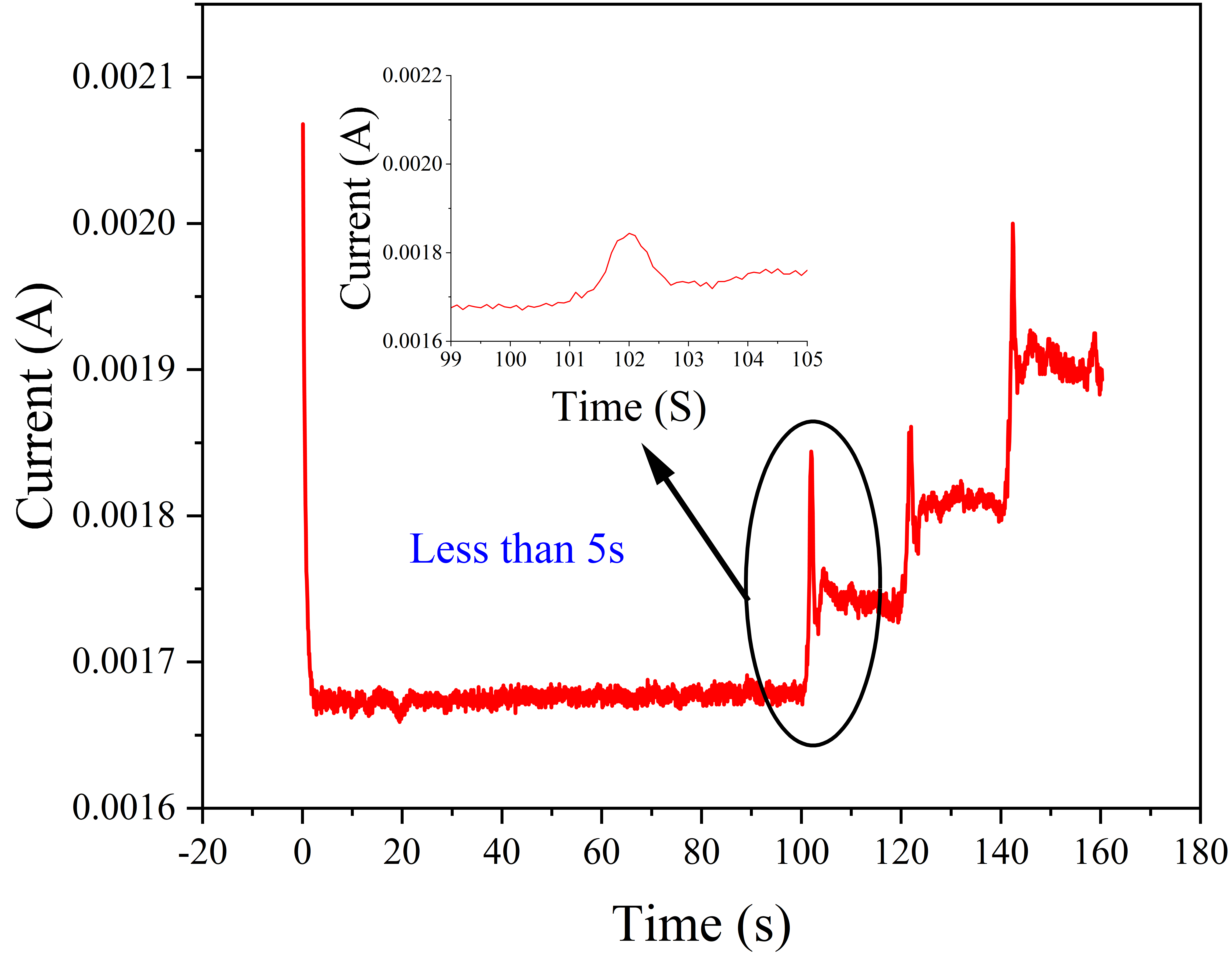


Fig.S4. The time response curve of the GF/Au/Ni(OH)_2_ composite fiber

150°

120°

90°

60°

30°

30°

Fig.S5. The schematics of the bending models.

As depicted in Table S2, when the fiber is bent at 30° and 60° for 100 times, the performance remains essentially unaffected (99.6% and 99.1%). Even when the fiber is bent at 90°, the performance can still be maintained at 97.9%. Nevertheless, when the fiber is bent at 120°, the performance begins to significantly decline (95.4%). However, when the fiber is bent at 150°, there is still 91.5% performance. Thus, the GF/Au/Ni(OH)2 electrode holds great potential for various applications..

Table S2 The performance of fiber sensor following 100 bending cycles at angles ranging from 30 to 150 degrees (30, 60, 90, 120, and 150 degrees, respectively)

| Angle of bending | 30° | 60° | 90° | 120° | 150° |
| --- | --- | --- | --- | --- | --- |
| I/I_0_ | 99.6% | 99.1% | 97.9% | 95.4% | 91.5% |

Table S3 The comparison on RSD and RE between commercial glucose meter and GF/Au/Ni(OH)_2_.

| Sample | Commercial glucose meter | |  | GF/Au/Ni(OH)_2_ | | |
| --- | --- | --- | --- | --- | --- | --- |
|  | Value mM | RSD |  | Value mM | RSD | RE |
| 1^#^ | 1.35 | 5.5% |  | 1.29 | 3.2% | 4.4% |
| 2^#^ | 0.60 | 0.5% |  | 0.57 | 1.5% | 5% |
| 3^#^ | 0.63 | 2.3% |  | 0.62 | 2.2% | 1.6% |


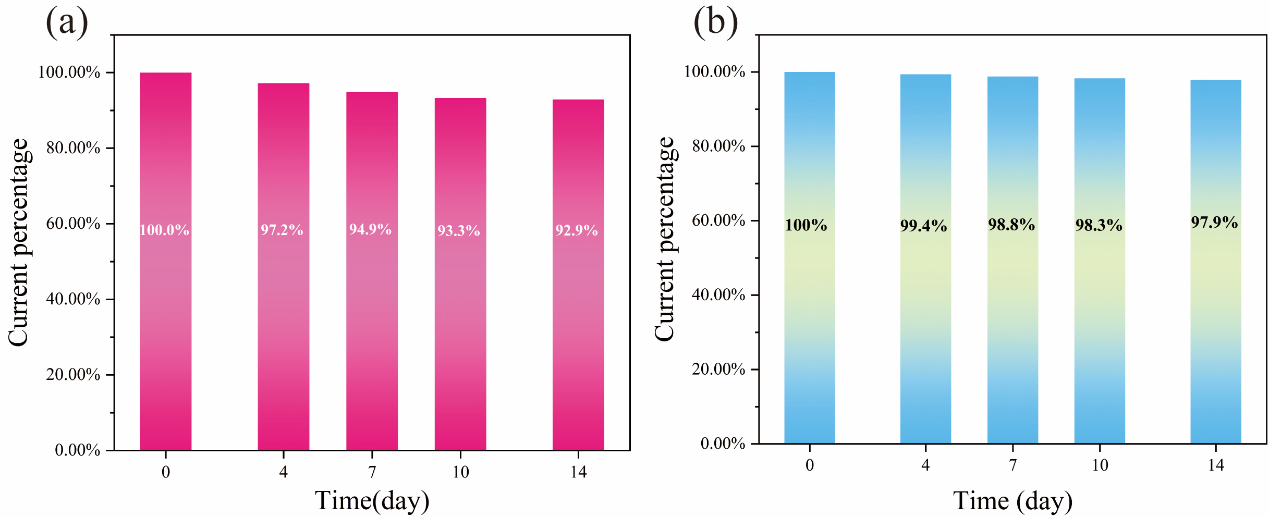


Fig.S6. (a)Current response variation of 1 mM glucose on GF/Au/Ni(OH)_2_ electrode versus storage time. (b)Current response variation of 1 mM glucose on GF/Au/Ni(OH)_2_ electrode versus storage time without humidity.


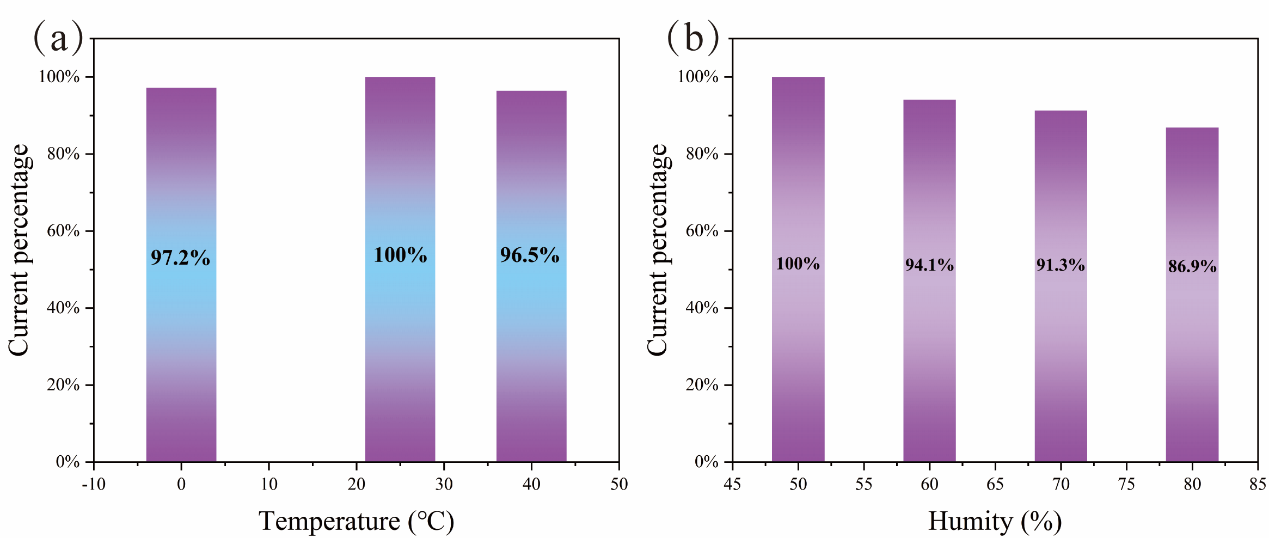


Fig.S7. (a) Current response of GF/Au/Ni(OH)_2_ electrode under different temperature storage conditions. (b) Current response of GF/Au/Ni(OH)_2_ electrode under different humidity storage conditions.

Three types of synthetic perspiration were evaluated utilizing GF/Au/Ni(OH)_2_ glucose sensors and a commercial blood glucose meter (Glucose meter 580, Yuwell, China). Each sample was measured three times, and the average glucose concentration was calculated. According to the Table S3, the relative standard deviation (RSD) of the prepared glucose sensor is below 4%, indicating its superior accuracy in measuring glucose concentration in sweat. The relative error (RE) falls within 5.0%, suggesting that the obtained results align well with those from commercially available sweat glucose meters. Therefore, our glucose sensor demonstrates exceptional reliability in measuring sweat glucose levels.

Table S4 The calculations of Muliken charges for glucose adsorption.

| Electrode materials | Muliken electric charge(e) |
| --- | --- |
| GF | 0.17 |
| GF/Au | 0.34 |
| GF/Au/Ni(OH)_2_ | 0.42 |

The detection process of glucose sensors is influenced by pH (Fig.S8) and temperature. To enhance the precision of measurements, it is recommended to concurrently utilize pH sensors and temperature sensors in order to ensure accurate results. Furthermore, the recently developed laser-induced graphene technology has the potential to form porous graphene structures, thereby increasing the specific surface area of graphene fibers and further boosting the sensitivity of this process.

**
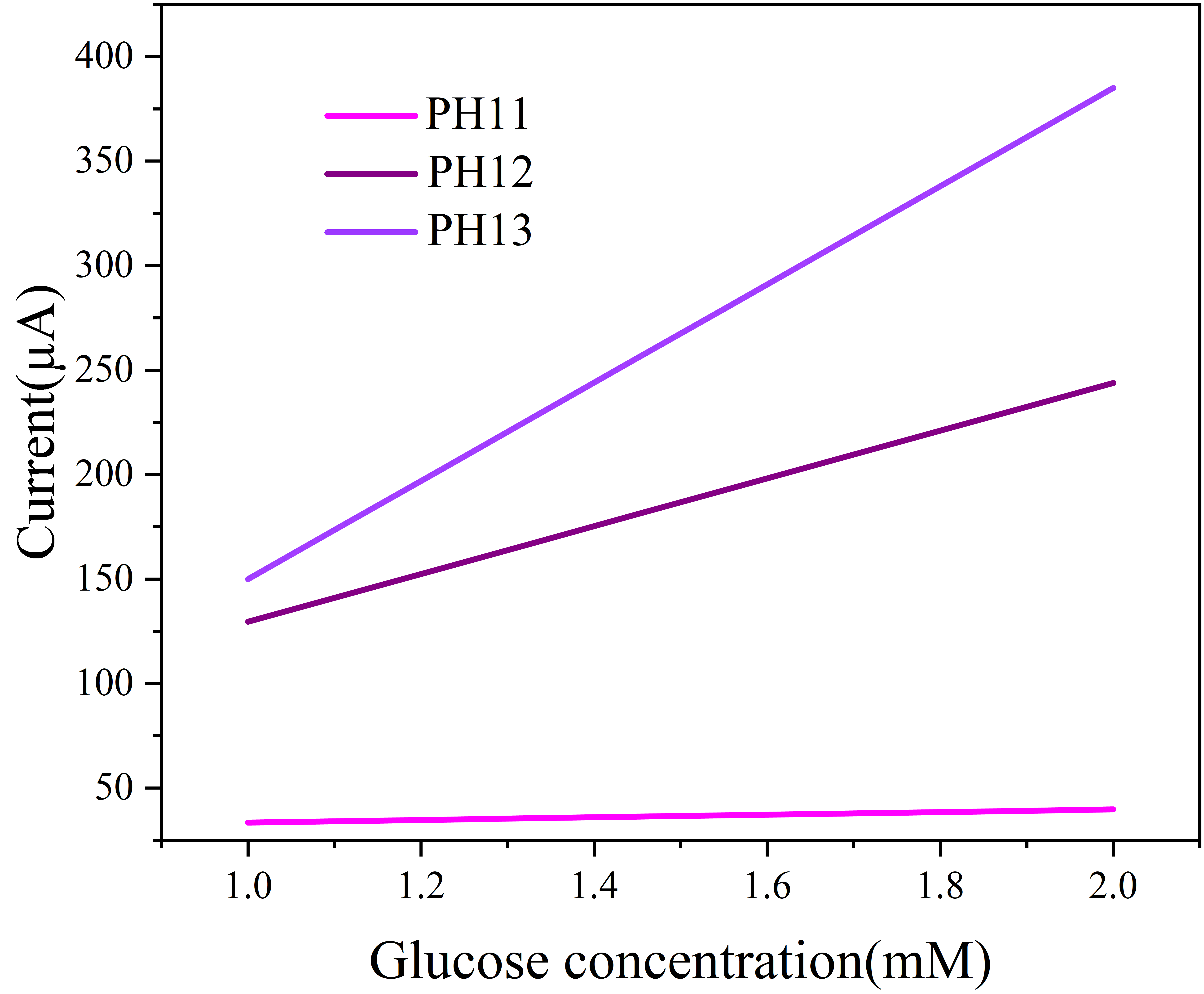
**

Fig.S8. The performance of GF/Au/Ni(OH)_2_ sensor under distinct pH level.

**Reference:**

[1] Tian Lu, Feiwu Chen, Multiwfn: A Multifunctional Wavefunction Analyzer, *J. Comput. Chem*. **2012**, 33(5), 580-592.
